# Association between Life’s simple 7 and rheumatoid arthritis in adult Americans: data from the National Health and nutrition examination survey

**DOI:** 10.3389/fpubh.2023.1251002

**Published:** 2023-11-29

**Authors:** Jie Wang, Fei Xing, Ning Sheng, Zhou Xiang

**Affiliations:** Department of Orthopaedics, Orthopaedic Research Institute, West China Hospital, Sichuan University, Chengdu, Sichuan, China

**Keywords:** Life’s simple 7, rheumatoid arthritis, cardiovascular disease, healthy lifestyle behaviors, the American Heart Association (AHA)

## Abstract

**Objective:**

The study aimed to investigate the relationship between Life’s Simple 7 (LS7) and the risk of rheumatoid arthritis (RA) in adult Americans.

**Methods:**

A total of 17,532 participants were included in this study. The association between LS7 and the risk of RA was assessed using a weighted logistic regression model, with odds ratios (ORs) and 95% confidence intervals (CIs) calculated. Moreover, the nonlinear relationship was further characterized through smooth curve fitting (SCF) and weighted generalized additive model (GAM) analysis.

**Results:**

After adjusting for all covariates, the weighted logistic regression model demonstrated that the LS7 was negatively correlated with the risk of RA. Compared to quintile 1 of LS7, the OR between the risk of RA and quartile 4 of LS7 (LS7.Q4) was 0.261 (95% CI, 0.203, 0.337) in males under 50 years old, while in females of the same age group, the OR was 0.183 (95% CI, 0.142, 0.234). For females aged between 50 and 70 years old, the OR between the risk of RA and LS7.Q4 was 0.313 (95% CI, 0.264, 0.371). In females aged 70 years or older, the OR between the risk of RA and LS7.Q4 was 0.632 (95% CI, 0.486, 0.822).

**Conclusion:**

This finding suggested the healthy lifestyle behaviors represented by LS7 have a negative association with RA. However, further prospective studies are needed to verify the causal relationship in the results.

## 1 Introduction

Rheumatoid arthritis (RA) is a systemic autoimmune disease characterized by symmetric polyarthritis, affecting approximately 1% of the global population (1). It can present as fever, swollen and painful joints, joint disc formation, cartilage degeneration, bone erosion, joint deformity, functional disability, and progressive disability (1). Furthermore, RA is often accompanied by other comorbidities such as cardiovascular disease (CVD), severe infections, respiratory diseases, osteoporosis, cancer and so on (2–4). Compared to the general population, RA patients have a lower quality of life, higher economic burden, and greater risk of mortality (2–4). Although studies have reported that the progression of RA is related to various genetic factors, environmental factors, and lifestyle habits, the exact cause of the disease is not yet fully understood (5, 6).

It is worth noting that the relationship between RA and CVD is particularly strong (7–10). Previous studies have shown that RA patients have a CVD risk that is approximately 48% higher than the general population (9). In addition, CVD has been identified as the primary cause of premature death and sudden deaths in RA patients, and approximately 50% of RA patients’ deaths can be attributed to CVD-related causes (7, 8). Therefore, thoroughly understanding the role of CVD-related risk factors in RA patients is crucial in the early identification, prevention, and treatment of RA (7). However, while studies have indicated that various CVD-related risk factors, such as hypertension, dyslipidemia, diabetes, or dietary quality are associated with RA, the results are still controversial. Some studies have found no significant differences in these factors between RA and non-RA subjects (7, 10–12). This phenomenon may be partially explained by the fact that the occurrence and progression of RA may be the result of multiple interacting factors, with inherent interactions among various factors themselves. Although a single factor may impact RA, its impact is limited and easily influenced. Therefore, when assessing the impact of CVD-related risk factors on RA, it may be more appropriate to integrate multiple relevant factors into a comprehensive evaluation index (7, 10).

In 2010, the American Heart Association (AHA) established an ideal cardiovascular health monitoring indicator called the Life’s Simple 7 (LS7) that focuses on seven health factors to prevent CVD. The seven factors are divided into three medical examination indicators, which include total serum cholesterol, blood pressure, and fasting blood glucose, and four behavioral factors, which include smoking, body mass index (BMI), physical activity, and diet (13). The LS7 has been utilized by the AHA to achieve the strategic goal of monitoring and improving the cardiovascular health of Americans until 2020 and beyond (13). Numerous studies have shown that individuals with higher LS7 scores tend to have a better quality of life, lower risk of CVD, and all-cause mortality (14, 15). Furthermore, the LS7 can also be used to assess the risk of non-CVD such as cancer (16), diabetes (17), depression (18), ocular diseases (19), and kidney disease (20).

However, to our knowledge, there are currently no studies that have assessed the relationship between the combination of health factors defined by LS7 and the incidence of RA. Therefore, this study aims to explore the association between LS7 and RA in adult Americans.

## 2 Materials and methods

### 2.1 Study population

**Figure 1:**
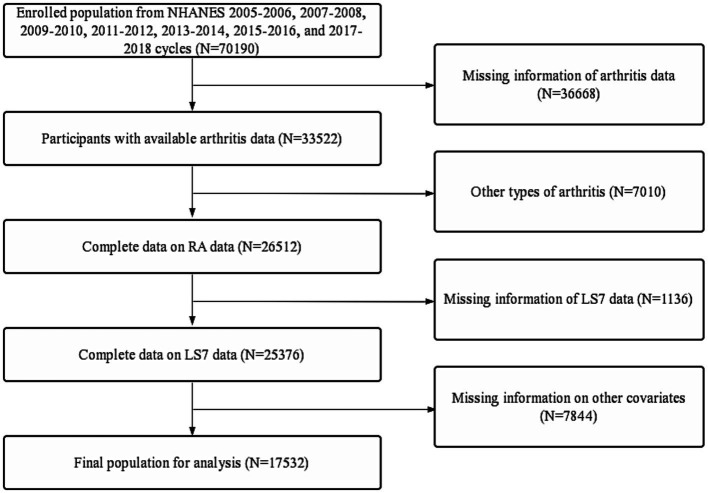
Flow diagram of inclusion criteria and exclusion criteria. NHANES, National Health and Nutrition Examination Survey; LS7, Life’s Simple 7; RA, rheumatoid arthritis.

This study utilized data from the National Health and Nutrition Examination Survey (NHANES) spanning from 2005 to 2018. NHANES gathered information from a diverse and nationally representative sample of the civilian population in the United States, using a multistage probability design. In addition, NHANES was overseen and approved by the ethical review board of the National Center for Health Statistics, and all participants provided written informed consent. The detailed inclusion and exclusion criteria for this study were presented in Figure 1.

### 2.2 Assessment of RA

RA was diagnosed by health professionals and relevant information was collected through a questionnaire. Specifically, participants were asked two questions related to arthritis. Firstly, they were asked, “Has a doctor or other health professional ever told you that you have arthritis?” Those who answered “yes” were then asked the second question: “What type of arthritis is it?” Participants who answered “RA” were included in the study. Interview data that was incomplete, as well as people who had other types of arthritis such as osteoarthritis and psoriatic arthritis, were excluded from the study to ensure the accuracy of the findings.

### 2.3 LS7 calculation

The calculation method of LS7 is based on the AHA guidelines, with seven indicators including blood pressure, total cholesterol, glycated hemoglobin (HbA1c), smoking, BMI, physical activity, and diet (21). Table 1 displayed the definition of poor (score 0), moderate (score 1), and ideal (score 2) levels of each indicator. Blood pressure was calculated as the average of three continuous measurements obtained at a mobile examination center (MEC). Total serum cholesterol in the NHANES database was measured via enzymatic methods, while HbA1c was measured using a Tosoh G7 analyzer (Tosoh, Tokyo, Japan) (18). Smoking status was identified through self-report, and BMI was calculated by trained health technicians using height and weight data. Physical activity was assessed through a questionnaire survey of the frequency and duration of moderate and high-intensity sports activities in the past 30 days. In addition, this study used the Healthy Eating Index-2015 (HEI-2015) to evaluate diet, and dietary data were obtained from two 24-h recall interviews conducted by NHANES. The first interview was conducted at a MEC, and the second interview was conducted via telephone 3 to 10 days after the first interview. The average of the two 24-h recall data was calculated and used as the dietary data for this study. The sum of the scores for all seven indicators is the final LS7 score.

**Table 1:** The Life’s Simple 7 scheme.

	Score		
	Poor (0)	Intermediate (1)	Ideal (2)
Blood pressure	Treated BP ≥140/90 mm Hg, and BP ≥140/90 mm Hg	SBP 120 to 139 mm Hg or DBP 80 to 89 mm Hg or treated to <120/80 mm Hg	<120/80 mm Hg, without BP-lowering meds
Total cholesterol	≥240 mg/dL	200 to 239 mg/dL or treated to <200 mg/dL	<200 mg/dL, without lipid-lowering medication
Glucose/diabetes	HbA1c >6.4%	HbA1c 5.7 to 6.4% or treated with insulin or oral meds to HbA1c <5.7%	HbA1c <5.7%, without meds
Smoking	Current smoker	Former smoker	Never smoker
Body weight	BMI ≥30 kg/m^2^	25 to 29.9 kg/m^2^	<25 kg/m^2^
Physical activity	No activity	1 to 149 min moderate/vigorous per week	≥150 min moderate/vigorous per week
Diet	HEI <50	HEI 50 to 80	HEI >80

BMI, body mass index; BP, blood pressure; SBP, systolic blood pressure; DBP, diastolic blood pressure; FPG, fasting plasma glucose; HbA1c, hemoglobin A1c; HEI, healthy eating index.

### 2.4 Covariates

Based on existing literature and clinical experience, this study selected covariates including age (<50 years, 50–70 years, ≥70 years), Sex (male, female), race (Mexican Americans, other Hispanic, non-Hispanic White, non-Hispanic Black, other race), educational level (less than 9th grade, 9–11th grade, high school, some college, college graduate), marital status (married, widowed, divorced, separated, never married, living with partner), and poverty income ratio (PIR) (<1, 1–3, ≥3), alcohol consumption (drink/d), and the estimated glomerular filtration rate (eGFR), which was calculated based on the Chronic Kidney Disease Epidemiology Collaboration (CKD-EPI) equation (22).

### 2.5 Statistical analysis

All analyses were conducted using the sampling weights according to the NHANES sampling criteria. Means and proportions were used to describe continuous and categorical variables, respectively. Student’s t-test was used to compare continuous variables between the RA group and the non-RA group, while chi-square test was used for categorical variables. We evaluated the association between LS7 and RA risk using a weighted logistic regression model, which calculated odds ratios (ORs) and 95% confidence intervals (CIs). Nonlinear relationships were characterized using smooth curve fitting (SCF) and weighted generalized additive models (GAMs). Additionally, we conducted subgroup analyses using a weighted logistic regression model based on age and sex. To ensure robustness of data analysis, LS7 values were classified into quartiles, and linear trend tests were conducted. The same steps were followed to evaluate the relationship between LS7 quartiles [(LS7.Q)] and RA risk. Model 1 was not adjusted for covariates. Model 2 was adjusted for age (if applicable), sex (if applicable), and race. In Model 3, covariate adjustment included age (if applicable), sex (if applicable), race, educational level, marital status, PIR, eGFR, and alcohol consumption. We also incorporated missing variables in the covariates as dummy variables and included other types of arthritis in Non-RA group to performed the sensitivity analysis. The ability of LS7 to identify RA was analyzed using the receiver operating characteristic (ROC) curve. The cut-off value for LS7 to identify RA was determined based on the maximum Youden index (sensitivity + specificity - 1). Additionally, the usefulness of the LS7 cut-off value in assessing RA was evaluated using sensitivity, specificity, positive predictive value (PPV), and negative predictive value (NPV).

All analyses were performed using R software (version 4.0.3) and EmpowerStats (version 2.0). A two-sided value of p less than 0.05 was set to determine statistical significance.

## 3 Results

### 3.1 Baseline characteristics of participants

Initially, data from 70,190 participants were collected by merging the continuous NHANES cycles from 2005–2006, 2007–2008, 2009–2010, 2011–2012, 2013–2014, 2015–2016, and 2017–2018. Participants with missing data on arthritis (*n* = 36,668) and arthritis other than RA (*n* = 7,010) were excluded, along with those with missing data on LS7 (*n* = 1,136) and other covariates (*n* = 7,844). The final analysis included 17,532 participants (Figure 1). Compared to the non-RA group, the RA group had a significantly higher prevalence of diabetes (10.5% vs. 20.0%, *p* < 0.001), hypertension (25.7% vs. 54.2%, *p* < 0.001), and CVD (5.1% vs. 18.9%, *p* < 0.001). Furthermore, the RA group had significantly lower LS7 values than the non-RA group (8.7 ± 2.3 vs. 7.3 ± 2.2, *p* < 0.001). The analysis of other variables revealed that RA patients were generally older, female, non-Hispanic white, overweight, impoverished, widowed, had lower levels of education, smoked more, drunk less, exercised less, had higher serum total cholesterol levels, and poorer kidney function (*p* < 0.05, Table 2).

**Table 2:** Weighted characteristics of the study population.

	Non-RA (N = 16,124)	RA (N = 1,408)	*p* value
Age (%)			<0.001
<50	65.9	22.9	
≥50, <70	27	52.8	
≥70	7.1	24.3	
Sex (%)			<0.001
male	50.6	38.4	
female	49.4	61.6	
Race (%)			<0.001
Mexican Americans	9.2	4.0	
other Hispanic	5.7	3.3	
non-Hispanic White	67.1	76.6	
non-Hispanic Black	10.6	10.3	
other race	7.5	5.9	
BMI (%)			<0.001
<25	31.9	23.1	
≥25, <30	33.8	32.9	
≥30	34.2	44.0	
PIR (%)			0.001
<1	12.9	15.2	
≥1, <3	34.8	37.9	
≥3	52.3	46.9	
Educational level (%)			<0.001
less than 9th grade	3.9	5.7	
9–11th grade	9.2	11.6	
high school	22.3	25.6	
some college	31.3	32.7	
college graduate	33.2	24.4	
Marital status (%)			<0.001
married	56.5	59.9	
widowed	3.1	12.2	
divorced	9.4	13.5	
separated	2.1	2.6	
never married	19.9	7.6	
living with partner	9.0	4.2	
CVD (%)			<0.001
No	94.9	81.1	
Yes	5.1	18.9	
Diabetes status (%)			<0.001
No	81.8	70.1	
Yes	10.5	20.0	
borderline	7.7	9.9	
Hypertension status (%)			<0.001
No	74.3	45.8	
Yes	25.7	54.2	
eGFR (mL/(min · 1.73 m^2^), %)			<0.001
<60	4.6	14	
≥60, <90	28.4	48.9	
≥90	67	37.1	
Smoking (%)			<0.001
Never	58.7	45.8	
Former	22.5	36	
Current	18.8	18.2	
Moderate or vigorous activity (%)			<0.001
No	38.5	49.2	
Yes	61.5	50.8	
Alcohol consumption (drink/d, mean ± SD)	1.5 ± 3.3	1.0 ± 2.5	<0.001
Total cholesterol (mg/dL)	193.5 ± 40.8	199.8 ± 42.6	<0.001
LS7	8.7 ± 2.3	7.3 ± 2.2	<0.001
HEI-2015			
Total Scores	50.5 ± 13.8	51.1 ± 13.9	0.221
Total Vegetables	3.0 ± 1.7	3.1 ± 1.7	0.623
Greens and Beans	1.6 ± 2.2	1.3 ± 2.0	<0.001
Total Fruits	1.9 ± 2.0	2.3 ± 2.1	<0.001
Whole Fruits	2.0 ± 2.2	2.3 ± 2.2	<0.001
Whole Grains	2.4 ± 3.2	2.7 ± 3.4	0.005
Dairy	5.1 ± 3.4	5.1 ± 3.4	0.893
Total Protein Foods	4.2 ± 1.3	4.1 ± 1.3	0.063
Seafood and Plant	2.3 ± 2.3	2.2 ± 2.2	0.055
Fatty Acids	5.0 ± 3.6	4.7 ± 3.6	0.017
Sodium	4.4 ± 3.5	4.7 ± 3.5	0.003
Refined Grains	6.1 ± 3.7	6.5 ± 3.6	0.002
Saturated Fats	5.9 ± 3.5	5.6 ± 3.6	0.010
Added Sugars	6.6 ± 3.4	6.5 ± 3.5	0.262

RA, rheumatoid arthritis; CVD, cardiovascular disease; HEI, healthy eating index; PIR, poverty income ratio; BMI, body mass index; eGFR, estimated glomerular filtration rate; SD, standard deviation; %, weighted percentage.

### 3.2 Association between LS7 and RA

#### 3.2.1 Total analyses

**Figure 2:**
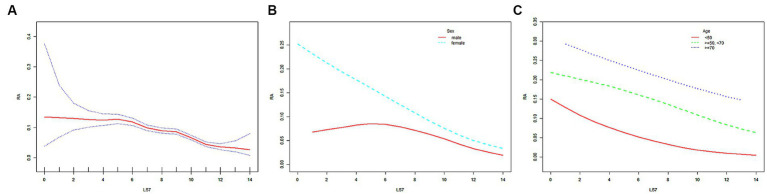
The SCF for the associations of LS7 with the risk of RA. Age (in **A,B**), sex (in **A,C**), race, educational level, marital status, PIR, eGFR, and alcohol consumption were adjusted. RA, rheumatoid arthritis; LS7, Life’s Simple 7; PIR, poverty income ratio; eGFR, estimated glomerular filtration rate; SD, standard deviation; %, weighted percentage. The vertical axis represented the probability of RA.

In the weighted logistic regression model, LS7 showed a negative correlation with RA risk in Model 1. This trend remained stable even after adjusting for confounding factors in Model 2 (age, sex, and race) and Model 3 (all covariates), as demonstrated in Table 3. When LS7 was divided into quartiles, ORs between the risk of RA and LS7 across quintiles 2 (LS7.Q2), 3 (LS7.Q3), and 4 (LS7.Q4) compared with quintile 1 (LS7.Q1) in Model 1 were 0.681 (95% CI, 0.624, 0.744), 0.664 (95% CI, 0.619, 0.712) and 0.364 (95% CI, 0.335, 0.397), respectively. After adjusting for covariates in Model 2, ORs between the risk of RA and LS7 across LS7.Q2, LS7.Q3, and LS7.Q4 were found to be 0.678 (95% CI, 0.621, 0.741), 0.653 (95% CI, 0.609, 0.701) and 0.354 (95% CI, 0.325, 0.386), respectively. Further adjusting for covariates in Model 3, the results showed that ORs between the risk of RA and LS7 across LS7.Q2, LS7.Q3, and LS7.Q4 were found to be 0.685 (95% CI, 0.627, 0.749), 0.692 (95% CI, 0.644, 0.744) and 0.398 (95% CI, 0.364, 0.435), respectively (Table 4). Notably, trend tests indicated a linear association between LS7 quartiles and RA diagnosis (*p* for trend <0.05, Table 4). The SCF and GAM models also exhibited similar trends, as depicted in Figure 2A.

**Table 3:** Association of the LS7 and the risk of RA.

	Male	Female	Total
Age < 50			
Model 1 β (95% CI) *p* value	0.826 (0.796, 0.856) <0.001	0.738 (0.715, 0.762) <0.001	0.775 (0.757, 0.794) <0.001
Model 2 β (95% CI) *p* value	0.812 (0.783, 0.843) <0.001	0.739 (0.716, 0.763) <0.001	0.770 (0.752, 0.789) <0.001
Model 3 β (95% CI) *p* value	0.813 (0.782, 0.845) <0.001	0.761 (0.735, 0.788) <0.001	0.790 (0.771, 0.811) <0.001
Age ≥ 50, <70			
Model 1 β (95% CI) *p* value	0.985 (0.958, 1.012) 0.267	0.839 (0.821, 0.858) <0.001	0.893 (0.878, 0.908) <0.001
Model 2 β (95% CI) *p* value	0.987 (0.960, 1.015) 0.350	0.832 (0.813, 0.851) <0.001	0.891 (0.876, 0.906) <0.001
Model 3 β (95% CI) *p* value	0.990 (0.961, 1.019) 0.480	0.856 (0.836, 0.877) <0.001	0.907 (0.891, 0.924) <0.001
Age ≥ 70			
Model 1 β (95% CI) *p* value	0.910 (0.864, 0.958) <0.001	0.889 (0.856, 0.923) <0.001	0.896 (0.869, 0.924) <0.001
Model 2 β (95% CI) *p* value	0.909 (0.863, 0.958) <0.001	0.894 (0.860, 0.929) <0.001	0.898 (0.871, 0.927) <0.001
Model 3 β (95% CI) *p* value	0.931 (0.881, 0.984) 0.011	0.908 (0.871, 0.946) <0.001	0.914 (0.885, 0.945) <0.001
Total			
Model 1 β (95% CI) *p* value	0.923 (0.904, 0.941) <0.001	0.819 (0.806, 0.833) <0.001	0.858 (0.847, 0.869) <0.001
Model 2 β (95% CI) *p* value	0.918 (0.900, 0.937) <0.001	0.815 (0.802, 0.829) <0.001	0.854 (0.843, 0.865) <0.001
Model 3 β (95% CI) *p* value	0.928 (0.909, 0.948) <0.001	0.834 (0.820, 0.849) <0.001	0.870 (0.858, 0.881) <0.001

Model 1: no covariates were adjusted.

Model 2: age (if applicable), sex (if applicable), and race were adjusted.

Model 3: age (if applicable), sex (if applicable), race, educational level, marital status, PIR, eGFR, and alcohol consumption were adjusted.

RA, rheumatoid arthritis; LS7, Life’s Simple 7; PIR, poverty income ratio; eGFR, estimated glomerular filtration rate; SD, standard deviation; %, weighted percentage.

**Table 4:** Association of the LS7.Q and the risk of RA.

	Male	Female	Total
Age < 50			
Model 1 β (95% CI) *p* value			
LS7.Q			
Q1	1	1	1
Q2	0.737 (0.562, 0.966) 0.027	0.796 (0.622, 1.017) 0.068	0.771 (0.643, 0.924) 0.005
Q3	0.656 (0.532, 0.809) <0.001	0.577 (0.475, 0.703) <0.001	0.616 (0.533, 0.710) <0.001
Q4	0.294 (0.231, 0.374) <0.001	0.140 (0.110, 0.177) <0.001	0.195 (0.165, 0.231) <0.001
Model 2 β (95% CI) *p* value			
LS7.Q			
Q1	1	1	1
Q2	0.689 (0.525, 0.904) 0.007	0.814 (0.636, 1.041) 0.101	0.758 (0.632, 0.910) 0.003
Q3	0.612 (0.496, 0.755) <0.001	0.592 (0.486, 0.721) <0.001	0.606 (0.525, 0.700) <0.001
Q4	0.266 (0.209, 0.339) <0.001	0.140 (0.111, 0.178) <0.001	0.187 (0.157, 0.221) <0.001
Model 3 β (95% CI) *p* value			
LS7.Q			
Q1	1	1	1
Q2	0.635 (0.481, 0.838) 0.001	0.808 (0.630, 1.036) 0.093	0.735 (0.611, 0.884) 0.001
Q3	0.596 (0.480, 0.741) <0.001	0.628 (0.514, 0.768) <0.001	0.640 (0.553, 0.741) <0.001
Q4	0.261 (0.203, 0.337) <0.001	0.183 (0.142, 0.234) <0.001	0.220 (0.184, 0.262) <0.001
P trend	<0.001	<0.001	<0.001
Age ≥ 50, <70			
Model 1 β (95% CI) *p* value			
LS7.Q			
Q1	1	1	1
Q2	0.540 (0.434, 0.671) <0.001	0.597 (0.512, 0.696) <0.001	0.584 (0.515, 0.661) <0.001
Q3	0.705 (0.601, 0.827) <0.001	0.648 (0.574, 0.731) <0.001	0.674 (0.612, 0.742) <0.001
Q4	1.005 (0.856, 1.180) 0.948	0.276 (0.236, 0.324) <0.001	0.492 (0.440, 0.550) <0.001
Model 2 β (95% CI) *p* value			
LS7.Q			
Q1	1	1	1
Q2	0.540 (0.434, 0.671) <0.001	0.591 (0.507, 0.690) <0.001	0.582 (0.514, 0.659) <0.001
Q3	0.702 (0.598, 0.824) <0.001	0.625 (0.553, 0.707) <0.001	0.669 (0.607, 0.736) <0.001
Q4	1.031 (0.876, 1.212) 0.716	0.262 (0.223, 0.307) <0.001	0.484 (0.433, 0.542) <0.001
Model 3 β (95% CI) *p* value			
LS7.Q			
Q1	1	1	1
Q2	0.524 (0.420, 0.654) <0.001	0.620 (0.530, 0.727) <0.001	0.596 (0.525, 0.677) <0.001
Q3	0.691 (0.587, 0.813) <0.001	0.694 (0.611, 0.789) <0.001	0.708 (0.642, 0.782) <0.001
Q4	1.061 (0.897, 1.256) 0.490	0.313 (0.264, 0.371) <0.001	0.540 (0.480, 0.607) <0.001
P trend	0.995	<0.001	<0.001
Age ≥ 70			
Model 1 β (95% CI) *p* value			
LS7.Q			
Q1	1	1	1
Q2	0.966 (0.736, 1.268) 0.804	0.692 (0.557, 0.859) <0.001	0.785 (0.663, 0.930) 0.005
Q3	0.751 (0.591, 0.955) 0.019	0.558 (0.461, 0.675) <0.001	0.625 (0.538, 0.725) <0.001
Q4	0.463 (0.316, 0.680) <0.001	0.553 (0.432, 0.709) <0.001	0.528 (0.429, 0.649) <0.001
Model 2 β (95% CI) *p* value			
LS7.Q			
Q1	1	1	1
Q2	0.967 (0.736, 1.270) 0.808	0.704 (0.566, 0.875) 0.002	0.792 (0.668, 0.940) 0.007
Q3	0.735 (0.578, 0.936) 0.012	0.567 (0.467, 0.687) <0.001	0.625 (0.538, 0.726) <0.001
Q4	0.467 (0.318, 0.686) <0.001	0.563 (0.438, 0.723) <0.001	0.533 (0.433, 0.655) <0.001
Model 3 β (95% CI) P value			
LS7.Q			
Q1	1	1	1
Q2	0.955 (0.721, 1.265) 0.748	0.723 (0.579, 0.904) 0.004	0.803 (0.676, 0.955) 0.013
Q3	0.782 (0.610, 1.004) 0.054	0.581 (0.475, 0.709) <0.001	0.652 (0.559, 0.760) <0.001
Q4	0.538 (0.361, 0.802) 0.002	0.632 (0.486, 0.822) <0.001	0.587 (0.473, 0.728) <0.001
P trend	0.002	<0.001	<0.001
Total			
Model 1 β (95% CI) P value			
LS7.Q			
Q1	1	1	1
Q2	0.701 (0.608, 0.809) <0.001	0.664 (0.594, 0.743) <0.001	0.681 (0.624, 0.744) <0.001
Q3	0.726 (0.649, 0.812) <0.001	0.624 (0.570, 0.683) <0.001	0.664 (0.619, 0.712) <0.001
Q4	0.623 (0.549, 0.707) <0.001	0.249 (0.222, 0.280) <0.001	0.364 (0.335, 0.397) <0.001
Model 2 β (95% CI) P value			
LS7.Q			
Q1	1	1	1
Q2	0.698 (0.605, 0.805) <0.001	0.663 (0.592, 0.742) <0.001	0.678 (0.621, 0.741) <0.001
Q3	0.716 (0.640, 0.802) <0.001	0.611 (0.558, 0.670) <0.001	0.653 (0.609, 0.701) <0.001
Q4	0.604 (0.532, 0.685) <0.001	0.242 (0.215, 0.272) <0.001	0.354 (0.325, 0.386) <0.001
Model 3 β (95% CI) *p* value			
LS7.Q			
Q1	1	1	1
Q2	0.680 (0.589, 0.786) <0.001	0.685 (0.611, 0.767) <0.001	0.685 (0.627, 0.749) <0.001
Q3	0.738 (0.658, 0.827) <0.001	0.658 (0.599, 0.722) <0.001	0.692 (0.644, 0.744) <0.001
Q4	0.647 (0.568, 0.737) <0.001	0.281 (0.248, 0.318) <0.001	0.398 (0.364, 0.435) <0.001
P trend	<0.001	<0.001	<0.001

Model 1: no covariates were adjusted.

Model 2: age (if applicable), sex (if applicable), and race were adjusted.

Model 3: age (if applicable), sex (if applicable), race, educational level, marital status, PIR, eGFR, and alcohol consumption were adjusted.

RA, rheumatoid arthritis; LS7.Q, quartile of Life’s Simple 7; PIR, poverty income ratio; eGFR, estimated glomerular filtration rate; SD, standard deviation; %, weighted percentage.

#### 3.2.2 Subgroup analyses

After stratifying the participants by sex or age, the negative correlation trend between LS7 and RA risk remained robust, as shown in Table 3. The negative correlation was further confirmed by the SCF and GAM models (Figures 2B,C). In the male population, ORs between the risk of RA and LS7 across LS7.Q2, LS7.Q3, and LS7.Q4 compared with LS7.Q1 in Model 1 were 0.701 (95% CI, 0.608, 0.809), 0.726 (95% CI, 0.649, 0.812) and 0.623 (95% CI, 0.549, 0.707), respectively. After adjusting for covariates, ORs between the risk of RA and LS7 across LS7.Q2, LS7.Q3, and LS7.Q4 in Model 2 were 0.698 (95% CI, 0.605, 0.805), 0.716 (95% CI, 0.640, 0.802) and 0.604 (95% CI, 0.532, 0.685), respectively. Further adjusting for covariates in Model 3, ORs between the risk of RA and LS7 across LS7.Q2, LS7.Q3, and LS7.Q4 were 0.680 (95% CI, 0.589, 0.786), 0.738 (95% CI, 0.658, 0.827) and 0.647 (95% CI, 0.568, 0.737), respectively (Table 4). In the female population, ORs between the risk of RA and LS7 across LS7.Q2, LS7.Q3, and LS7.Q4 in Model 1 were 0.664 (95% CI, 0.594, 0.743), 0.624 (95% CI, 0.570, 0.683) and 0.249 (95% CI, 0.222, 0.280), respectively. After adjusting for covariates, ORs between the risk of RA and LS7 across LS7.Q2, LS7.Q3, and LS7.Q4 in Model 2 were 0.663 (95% CI, 0.592, 0.742), 0.611 (95% CI, 0.558, 0.670) and 0.242 (95% CI, 0.215, 0.272), respectively. Further adjusting for covariates in Model 3, ORs between the risk of RA and LS7 across LS7.Q2, LS7.Q3, and LS7.Q4 were 0.685 (95% CI, 0.611, 0.767), 0.658 (95% CI, 0.599, 0.722) and 0.281 (95% CI, 0.248, 0.318), respectively (Table 4).

In the participants aged below 50 years, ORs between the risk of RA and LS7 across LS7.Q2, LS7.Q3, and LS7.Q4 in Model 1 were 0.771 (95% CI, 0.643, 0.924), 0.616 (95% CI, 0.533, 0.710) and 0.195 (95% CI, 0.165, 0.231), respectively. After adjusting for covariates, ORs between the risk of RA and LS7 across LS7.Q2, LS7.Q3, and LS7.Q4 in Model 2 were 0.758 (95% CI, 0.632, 0.910), 0.606 (95% CI, 0.525, 0.700) and 0.187 (95% CI, 0.157, 0.221), respectively. Further adjusting for covariates in Model 3, ORs between the risk of RA and LS7 across LS7.Q2, LS7.Q3, and LS7.Q4 were 0.735 (95% CI, 0.611, 0.884)), 0.640 (95% CI, 0.553, 0.741) and 0.220 (95% CI, 0.184, 0.262), respectively (Table 4). In the participants aged between 50 and 70 years, ORs between the risk of RA and LS7 across LS7.Q2, LS7.Q3, and LS7.Q4 in Model 1 were 0.584 (95% CI, 0.515, 0.661), 0.674 (95% CI, 0.612, 0.742) and 0.492 (95% CI, 0.440, 0.550), respectively. After adjusting for covariates, ORs between the risk of RA and LS7 across LS7.Q2, LS7.Q3, and LS7.Q4 in Model 2 were 0.582 (95% CI, 0.514, 0.659), 0.669 (95% CI, 0.607, 0.736) and 0.484 (95% CI, 0.433, 0.542), respectively. Further adjusting for covariates in Model 3, ORs between the risk of RA and LS7 across LS7.Q2, LS7.Q3, and LS7.Q4 were 0.596 (95% CI, 0.525, 0.677), 0.708 (95% CI, 0.642, 0.782) and 0.540 (95% CI, 0.480, 0.607), respectively (Table 4). In the participants aged 70 years or older, ORs between the risk of RA and LS7 across LS7.Q2, LS7.Q3, and LS7.Q4 in Model 1 were 0.785 (95% CI, 0.663, 0.930), 0.625 (95% CI, 0.538, 0.725) and 0.528 (95% CI, 0.429, 0.649), respectively. After adjusting for covariates, ORs between the risk of RA and LS7 across LS7.Q2, LS7.Q3, and LS7.Q4 in Model 2 were 0.792 (95% CI, 0.668, 0.940), 0.625 (95% CI, 0.538, 0.726) and 0.533 (95% CI, 0.433, 0.655), respectively. Further adjusting for covariates in Model 3, ORs between the risk of RA and LS7 across LS7.Q2, LS7.Q3, and LS7.Q4 were 0.803 (95% CI, 0.676, 0.955), 0.652 (95% CI, 0.559, 0.760) and 0.587 (95% CI, 0.473, 0.728), respectively (Table 4).

**Figure 3:**
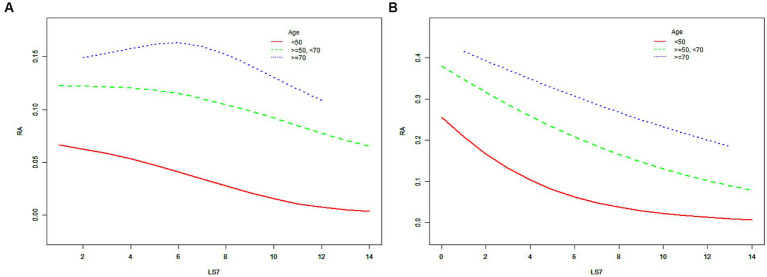
The SCF for the associations of LS7 with the risk of RA when the participants were further cross-stratified by age and sex. **(A)** male; **(B)** female. Race, educational level, marital status, PIR, eGFR, and alcohol consumption were adjusted. RA, rheumatoid arthritis; LS7, Life’s Simple 7; PIR, poverty income ratio; eGFR, estimated glomerular filtration rate; SD, standard deviation; %, weighted percentage. The vertical axis represented the probability of RA.

When the participants were further cross-stratified by age and sex, the negative association between LS7 and RA risk was mainly presented in males under 50 years old, while it was gratifying that this negative correlation is significant in female populations of all age groups (Table 3). The results of the SCF and GAM models in Figure 3 further characterize this relationship. After adjusting for all covariates, ORs between the risk of RA and LS7.Q4 in Model 3 were also compared for different populations. In males under 50 years old, the OR between the risk of RA and LS7.Q4 was 0.261 (95% CI, 0.203, 0.337), while in females of the same age group, the OR was 0.183 (95% CI, 0.142, 0.234). For females aged between 50 and 70 years old, the OR between the risk of RA and LS7.Q4 was 0.313 (95% CI, 0.264, 0.371). In females aged 70 years or older, the OR between the risk of RA and LS7.Q4 was 0.632 (95% CI, 0.486, 0.822). The robustness of the negative correlation between LS7 and RA was validated by the results of the sensitivity analysis (Appendix 1 and 2). All *p*-values for trend were < 0.05 according to Table 4.

#### 3.2.3 ROC curve

**Figure 4:**
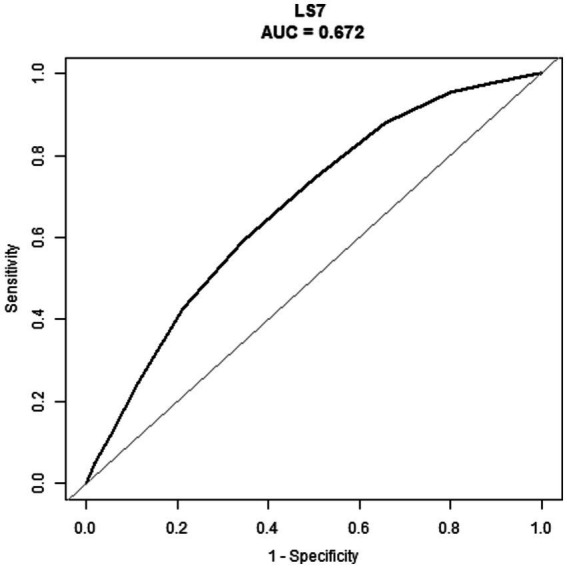
ROC of the LS7 to detect the risk of RA. RA, rheumatoid arthritis; LS7, Life’s Simple 7; ROC, receiver operating characteristic; AUC, area under the curve.

The ability of LS7 to detect RA was assessed using ROC curve analysis. As shown in the Figure 4, the area under the curve (AUC) of the ROC curve was 0.672 (95% CI, 0.659, 0.686). The LS7 score identified a cutoff value of 7.5 based on the highest Youden index, with a sensitivity of 59.1% and specificity of 65.6%. The PPV and NPV were 13.0 and 94.8%, respectively.

## 4 Discussion

Based on a representative sample of adult Americans in the NHANES database, we found a negative correlation between LS7 scores and the risk of RA. Interestingly, the negative correlation was more pronounced in males of lower (under 50 years) age groups, while in females, this correlation held true across all age groups. Our results imply that a lifestyle reflective of LS7 may confer protective effects against RA, thus highlighting the importance of lifestyle choices in the prevention of this disease. In addition, when the LS7 score was determined to have a cutoff value of 7.5 based on the highest Youden index, the NPV in this study reached as high as 94.8%. This demonstrates its excellent screening value for identifying RA-negative patients. To our knowledge, this study is the first to explore the potential connection between LS7-based lifestyle and RA risk and provides novel insights into the role of lifestyle in disease prevention.

Previous researches have linked individual components of LS7, such as smoking, obesity, diabetes, hypertension, high cholesterol, and less physical activity with an increased risk of RA (5, 23–27). Similarly, our study found that RA patients had a higher prevalence of diabetes, hypertension, overweight, elevated serum cholesterol, more smoking history, and lower physical activity levels compared to those without RA. Additionally, multiple studies have established shared mechanisms between RA and CVD, including inflammation mediators, changes in lipoprotein function and composition, peptide/protein modifications, increased oxidative stress, subsequent immune response, and endothelial dysfunction (7, 8, 28). CVD-related risk factors can impact RA through these shared mechanisms. For example, environmental factors like smoking, obesity, diabetes, and physical inactivity can induce immune dysfunction in susceptible individuals, resulting in increased production of pro-inflammatory cytokines such as interleukin-8 (IL-8), IL-17A, and tumor necrosis factor-alpha by inflammatory cells, and subsequent excessive production of neutrophil extracellular traps (NETs) (29, 30). Although NETs are antimicrobial structures made up of lysates and granule proteins from activated neutrophils, excessive formation of NETs can result in damage to vital organs, including the cardiovascular system, and increase the risk of immune-related diseases such as RA (29, 30). Therefore, the association between comprehensive LS7 scores and RA may be not surprising.

In fact, the high score of LS7 represents a healthy and upward lifestyle (31). Its significance for RA is not limited to its role in the origin of RA, but also in improving the quality of life of RA patients (23–26, 32–34). Numerous observational studies have highlighted the benefits of quitting smoking in mitigating RA-related outcomes, which has been supported by animal studies (32, 33). For instance, Donate et al. demonstrated that smoking can activate the aryl hydrocarbon receptor on Th17 cells in RA patients, thereby upregulating miR-132 and inhibiting the induction of cyclooxygenase-2, resulting in worsened arthritis inflammation and bone destruction (32). Recent toxic mechanism study conducted by Heluany et al. has also demonstrated that smoking can exacerbate joint symptoms, lung inflammation and lung metallothionein expression, and cause toxic damage to splenocytes by activating the nicotine/α7 nicotinic acetylcholine receptor pathway (33). Regarding physical exercise, many randomized trials have emphasized its benefits for RA patients’ pain and disability (34). Meanwhile, literature reviews conducted by Metsios et al. and Verhoeven et al. have further highlighted the benefits of physical exercise, including improvement in self-esteem, reduction in depressive symptoms, better sleep quality, and reduced pain, and recommended that RA patients regularly engage in moderate aerobic exercise (25, 26). In addition, factors such as BMI, blood glucose, blood pressure, and total cholesterol are highly correlated with metabolic syndrome. As the crossroads of RA and related CVD, it is well known that improving metabolic syndrome benefits the progression of RA and related CVD diseases (23, 24). Moreover, extensive prior research has emphasized the importance of maintaining a healthy and balanced diet for individuals with RA (35–38). Several systematic reviews have indicated that supplementing with foods containing polyunsaturated fatty acids, vitamin D, quercetin, and probiotics containing lactobacillus can reduce RA disease activity and decrease the failure rate of drug therapy, providing a protective effect against the development of RA. Conversely, studies have suggested that RA patients should limit their consumption of red meat and sodium (35–38). Evidence indicated that the underlying mechanism by which dietary factors impact RA may be associated with alterations in gut microbiota (39, 40).

This study has several strengths. Firstly, it is the first study to investigate the relationship between the cumulative effects of CVD-related risk factors, as represented by LS7, and RA risk. Secondly, the study leveraged a large, nationally representative database with standardized data collection protocols, which reduces potential biases. Thirdly, the study conducted subgroup analyses based on sex and age groups and classified LS7 into quartiles, adding to the robustness of the data analysis. However, this study also has limitations. Firstly, the cross-sectional nature of the data means that there may be insufficient evidence to infer causality. Secondly, data collection methods, such as questionnaires and interviews, may introduce recall bias. Thirdly, although the study adjusted for covariates, unmeasured confounding factors may still exist. Finally, this study did not investigate the correlation between LS7 and biomarkers for RA, such as rheumatoid factor, as there was insufficient data available. Furthermore, it is worth noting that this study did not evaluate important indicators of RA severity and physical function, such as the clinical disease activity index, disease activity score with 28-joint count, or simplified disease activity index (4). This emphasizes the importance of conducting future prospective studies to address this gap in knowledge.

## 5 Conclusion

In conclusion, the results of this study indicated that adult Americans with a higher LS7 have a lower risk of RA. This finding suggested a negative association between the healthy lifestyle behaviors represented by LS7 with RA. However, further prospective studies are needed to verify the causal relationship in the results.

## Data availability statement

The raw data supporting the conclusions of this article will be made available by the authors, without undue reservation.

## Ethics statement

NHANES was approved by the National Center for Health Statistics Research Ethics Review Board.

## Author contributions

JW, FX, and NS: conceptualization and investigation. JW: methodology, analysis, and writing original draft. JW, FX, NS, and ZX: writing—review and editing. All authors contributed to the article and approved the submitted version.
