# Supplementary material for: Association between Life’s simple 7 and rheumatoid arthritis in adult Americans: data from the National Health and nutrition examination survey
Source: Front Public Health. 2023 Nov 29;11:1251002. doi: 10.3389/fpubh.2023.1251002 (PMC10716198; doi:10.3389/fpubh.2023.1251002)
Supplement: Supplementary file 1 [file Table_1.DOCX]

**Appendix 1 Association of the LS7 and the risk of RA**

|  | **Male** | **Female** | **Total** |
| --- | --- | --- | --- |
| **Age <50** |  |  |  |
| Model 1  β (95% CI) P value | 0.863 (0.837, 0.890) <0.001 | 0.815 (0.795, 0.836) <0.001 | 0.834 (0.818, 0.851) <0.001 |
| Model 2  β (95% CI) P value | 0.852 (0.825, 0.879) <0.001 | 0.814 (0.793, 0.835) <0.001 | 0.829 (0.813, 0.846) <0.001 |
| Model 3  β (95% CI) P value | 0.842 (0.810, 0.875) <0.001 | 0.799 (0.772, 0.827) <0.001 | 0.819 (0.799, 0.840) <0.001 |
| **Age** ≥**50, <70** |  |  |  |
| Model 1  β (95% CI) P value | 0.995 (0.971, 1.019) 0.655 | 0.914 (0.897, 0.931) <0.001 | 0.943 (0.930, 0.957) <0.001 |
| Model 2  β (95% CI) P value | 0.996 (0.972, 1.021) 0.760 | 0.913 (0.896, 0.931) <0.001 | 0.944 (0.930, 0.958) <0.001 |
| Model 3  β (95% CI) P value | 1.036 (1.006, 1.067) 0.020 | 0.932 (0.911, 0.953) <0.001 | 0.967 (0.950, 0.984) <0.001 |
| **Age** ≥**70** |  |  |  |
| Model 1  β (95% CI) P value | 0.970 (0.933, 1.009) 0.127 | 0.957 (0.930, 0.984) 0.002 | 0.961 (0.939, 0.983) <0.001 |
| Model 2  β (95% CI) P value | 0.970 (0.932, 1.009) 0.127 | 0.961 (0.934, 0.989) 0.006 | 0.964 (0.942, 0.987) 0.002 |
| Model 3  β (95% CI) P value | 0.968 (0.918, 1.020) 0.217 | 0.962 (0.928, 0.998) 0.038 | 0.967 (0.939, 0.995) 0.024 |
| **Total** |  |  |  |
| Model 1  β (95% CI) P value | 0.949 (0.933, 0.965) <0.001 | 0.895 (0.884, 0.908) <0.001 | 0.915 (0.905, 0.925) <0.001 |
| Model 2  β (95% CI) P value | 0.946 (0.929, 0.962) <0.001 | 0.894 (0.882, 0.907) <0.001 | 0.913 (0.903, 0.923) <0.001 |
| Model 3  β (95% CI) P value | 0.963 (0.943, 0.984) <0.001 | 0.899 (0.884, 0.914) <0.001 | 0.921 (0.909, 0.933) <0.001 |

Model 1: no covariates were adjusted.

Model 2: age (if applicable), sex (if applicable), and race were adjusted.

Model 3: age (if applicable), sex (if applicable), race, educational level, marital status, PIR, eGFR, and alcohol consumption were adjusted.

RA, rheumatoid arthritis; LS7, Life's Simple 7; PIR, poverty income ratio; eGFR, estimated glomerular filtration rate; SD, standard deviation; %, weighted percentage

**Appendix 2 Association of the LS7.Q and the risk of RA**

|  | **Male** | **Female** | **Total** |
| --- | --- | --- | --- |
| **Age <50** | | | |
| **Model 1**  **β (95% CI) P value** |  |  |  |
| **LS7.Q** |  |  |  |
| Q1 | 1 | 1 | 1 |
| Q2 | 0.695 (0.552, 0.876) 0.002 | 0.701 (0.568, 0.865) <0.001 | 0.699 (0.598, 0.817) <0.001 |
| Q3 | 0.660 (0.532, 0.819) <0.001 | 0.703 (0.580, 0.852) <0.001 | 0.686 (0.595, 0.792) <0.001 |
| Q4 | 0.311 (0.243, 0.396) <0.001 | 0.192 (0.153, 0.240) <0.001 | 0.234 (0.198, 0.276) <0.001 |
| **Model 2**  **β (95% CI) P value** |  |  |  |
| **LS7.Q** |  |  |  |
| Q1 | 1 | 1 | 1 |
| Q2 | 0.673 (0.534, 0.848) <0.001 | 0.700 (0.567, 0.864) <0.001 | 0.690 (0.590, 0.806) <0.001 |
| Q3 | 0.624 (0.503, 0.774) <0.001 | 0.705 (0.582, 0.855) <0.001 | 0.673 (0.583, 0.777) <0.001 |
| Q4 | 0.285 (0.223, 0.364) <0.001 | 0.190 (0.152, 0.238) <0.001 | 0.224 (0.190, 0.265) <0.001 |
| **Model 3**  **β (95% CI) P value** |  |  |  |
| **LS7.Q** |  |  |  |
| Q1 | 1 | 1 | 1 |
| Q2 | 0.693 (0.527, 0.913) <0.001 | 0.668 (0.519, 0.860) 0.002 | 0.686 (0.570, 0.825) <0.001 |
| Q3 | 0.626 (0.484, 0.809) <0.001 | 0.616 (0.486, 0.781) <0.001 | 0.641 (0.540, 0.762) <0.001 |
| Q4 | 0.276 (0.206, 0.368) <0.001 | 0.185 (0.140, 0.246) <0.001 | 0.227 (0.185, 0.277) <0.001 |
| **P trend** | <0.001 | <0.001 | <0.001 |
| **Age** ≥**50, <70** | | | |
| **Model 1**  **β (95% CI) P value** |  |  |  |
| **LS7.Q** |  |  |  |
| Q1 | 1 | 1 | 1 |
| Q2 | 0.762 (0.653, 0.889) <0.001 | 0.722 (0.641, 0.812) <0.001 | 0.736 (0.670, 0.808) <0.001 |
| Q3 | 0.776 (0.666, 0.906) 0.001 | 0.806 (0.718, 0.905) <0.001 | 0.795 (0.725, 0.872) <0.001 |
| Q4 | 1.192 (1.015, 1.400) 0.032 | 0.430 (0.369, 0.501) <0.001 | 0.676 (0.606, 0.754) <0.001 |
| Model 2  β (95% CI) P value |  |  |  |
| **LS7.Q** |  |  |  |
| Q1 | 1 | 1 | 1 |
| Q2 | 0.763 (0.654, 0.891) <0.001 | 0.728 (0.646, 0.819) <0.001 | 0.739 (0.672, 0.811) <0.001 |
| Q3 | 0.783 (0.671, 0.914) 0.002 | 0.805 (0.717, 0.905) <0.001 | 0.798 (0.728, 0.876) <0.001 |
| Q4 | 1.205 (1.025, 1.418) 0.024 | 0.430 (0.368, 0.501) <0.001 | 0.679 (0.609, 0.759) <0.001 |
| Model 3  β (95% CI) P value |  |  |  |
| **LS7.Q** |  |  |  |
| Q1 | 1 | 1 | 1 |
| Q2 | 0.832 (0.690, 1.005) 0.056 | 0.741 (0.646, 0.848) <0.001 | 0.772 (0.692, 0.861) <0.001 |
| Q3 | 0.843 (0.699, 1.018) 0.076 | 0.877 (0.766, 1.004) 0.057 | 0.859 (0.771, 0.958) 0.006 |
| Q4 | 1.495 (1.230, 1.817) <0.001 | 0.491 (0.411, 0.586) <0.001 | 0.790 (0.696, 0.897) <0.001 |
| **P trend** | <0.001 | <0.001 | 0.006 |
| **Age** ≥**70** | | | |
| **Model 1**  **β (95% CI) P value** |  |  |  |
| **LS7.Q** |  |  |  |
| Q1 | 1 | 1 | 1 |
| Q2 | 0.895 (0.727, 1.102) 0.297 | 0.992 (0.856, 1.151) 0.919 | 0.958 (0.849, 1.081) 0.488 |
| Q3 | 0.808 (0.646, 1.011) 0.062 | 0.827 (0.699, 0.979) 0.027 | 0.821 (0.718, 0.939) 0.004 |
| Q4 | 0.785 (0.582, 1.059) 0.113 | 0.712 (0.567, 0.893) 0.003 | 0.738 (0.616, 0.884) <0.001 |
| **Model 2**  **β (95% CI) P value** |  |  |  |
| **LS7.Q** |  |  |  |
| Q1 | 1 | 1 | 1 |
| Q2 | 0.901 (0.731, 1.110) 0.327 | 1.003 (0.864, 1.164) 0.971 | 0.967 (0.857, 1.092) 0.589 |
| Q3 | 0.806 (0.643, 1.010) 0.060 | 0.843 (0.712, 0.999) 0.049 | 0.831 (0.726, 0.952) 0.008 |
| Q4 | 0.786 (0.582, 1.062) 0.117 | 0.730 (0.581, 0.918) 0.007 | 0.752 (0.626, 0.902) 0.002 |
| **Model 3**  **β (95% CI) P value** |  |  |  |
| **LS7.Q** |  |  |  |
| Q1 | 1 | 1 | 1 |
| Q2 | 0.924 (0.711, 1.200) 0.552 | 0.964 (0.803, 1.159) 0.699 | 0.987 (0.851, 1.146) 0.868 |
| Q3 | 0.950 (0.724, 1.247) 0.711 | 0.733 (0.594, 0.905) 0.004 | 0.839 (0.712, 0.988) 0.036 |
| Q4 | 0.605 (0.399, 0.916) 0.018 | 0.851 (0.657, 1.103) 0.223 | 0.778 (0.627, 0.967) 0.024 |
| **P trend** | 0.081 | 0.014 | 0.004 |
| **Total** | | | |
| **Model 1**  **β (95% CI) P value** |  |  |  |
| **LS7.Q** |  |  |  |
| Q1 | 1 | 1 | 1 |
| Q2 | 0.792 (0.710, 0.884) <0.001 | 0.805 (0.739, 0.876) <0.001 | 0.800 (0.748, 0.855) <0.001 |
| Q3 | 0.783 (0.702, 0.873) <0.001 | 0.821 (0.755, 0.894) <0.001 | 0.806 (0.754, 0.862) <0.001 |
| Q4 | 0.739 (0.653, 0.836) <0.001 | 0.368 (0.329, 0.411) <0.001 | 0.494 (0.455, 0.536) <0.001 |
| **Model 2**  **β (95% CI) P value** |  |  |  |
| **LS7.Q** |  |  |  |
| Q1 | 1 | 1 | 1 |
| Q2 | 0.787 (0.705, 0.878) <0.001 | 0.807 (0.741, 0.878) <0.001 | 0.798 (0.746, 0.854) <0.001 |
| Q3 | 0.772 (0.692, 0.861) <0.001 | 0.817 (0.750, 0.890) <0.001 | 0.799 (0.747, 0.854) <0.001 |
| Q4 | 0.722 (0.638, 0.817) <0.001 | 0.366 (0.328, 0.410) <0.001 | 0.488 (0.449, 0.530) <0.001 |
| **Model 3**  **β (95% CI) P value** |  |  |  |
| **LS7.Q** |  |  |  |
| Q1 | 1 | 1 | 1 |
| Q2 | 0.846 (0.741, 0.966) 0.013 | 0.821 (0.743, 0.907) <0.001 | 0.829 (0.766, 0.898) <0.001 |
| Q3 | 0.855 (0.750, 0.975) 0.019 | 0.813 (0.735, 0.900) <0.001 | 0.827 (0.763, 0.896) <0.001 |
| Q4 | 0.810 (0.699, 0.940) 0.005 | 0.403 (0.354, 0.460) <0.001 | 0.538 (0.488, 0.593) <0.001 |
| **P trend** | 0.014 | <0.001 | <0.001 |

Model 1: no covariates were adjusted.

Model 2: age (if applicable), sex (if applicable), and race were adjusted.

Model 3: age (if applicable), sex (if applicable), race, educational level, marital status, PIR, eGFR, and alcohol consumption were adjusted.

RA, rheumatoid arthritis; LS7.Q, quartile of Life's Simple 7; PIR, poverty income ratio; eGFR, estimated glomerular filtration rate; SD, standard deviation; %, weighted percentage
